# Effects of moving cupping therapy for plaque psoriasis: study protocol for a randomized multicenter clinical trial

**DOI:** 10.1186/s13063-020-4155-0

**Published:** 2020-02-26

**Authors:** Meng Xing, Xiaoning Yan, Suqing Yang, Linge Li, Liping Gong, Hongxia Liu, Rong Xu, Jie Chen, Luo Ying, Yiding Zhao, Yuepeng An, Yang Liu, Gang Huang, Fei Guo, Qingfeng Yin, Ruiping Wang, Bin Li, Xin Li

**Affiliations:** 1 Department of Dermatology, Yueyang Hospital of Integrated Traditional Chinese and Western Medicine, Shanghai University of Traditional Chinese Medicine, Shanghai, 200437 China; 2 Department of Dermatology, Shaanxi Traditional Chinese Medicine Hospital, Shaanxi, 710003 China; 3 Department of Dermatology, First Affiliated Hospital of Heilongjiang University of Traditional Chinese Medicine, Harbin, 150040 Heilongjiang China; 4 Department of Dermatology, Shijiazhuang Hospital of Traditional Chinese Medicine, Shijiazhuang, 050051 China; 5 Department of Dermatology, The Affiliated Hospital of Jiangxi University of Traditional Chinese Medicine, Nanchang, 330006 China; 6 Department of Dermatology, Hospital of Traditional Chinese Medicine, Xinjiang Medicine University, Xinjiang, 830000 China; 7 Jiangsu Famous Medical Technology Co. Ltd, Nanjing University of Traditional Chinese Medicine, Nanjing, 210029 China; 8 Office of Clinical Medical Research Center, Yueyang Hospital of Integrated Traditional Chinese and Western Medicine, Shanghai University of Traditional Chinese Medicine, Shanghai, 200437 China; 9 Shanghai Academy of Traditional Chinese Medicine, Shanghai, 201203 China

**Keywords:** Plaque psoriasis, Moving cupping, Protocol, Randomized controlled trial

## Abstract

**Background:**

It is difficult to achieve a balance among safety, efficacy, and cost for the clinical treatment of plaque psoriasis. The current treatment of psoriasis often involves comprehensive therapy such as topical plasters, internal medicine, and phototherapy, which are expensive, and some of the drugs have serious side effects. Moving cupping is a type of cupping that has been used clinically for thousands of years in China. It has the advantage of being inexpensive and easy to perform. Therefore, it is widely used in public hospitals in China for psoriasis treatment. However, a comprehensive evaluation of the current clinical evidence of its efficacy is lacking. In this study, we aimed to evaluate the efficacy and safety of moving cupping to treat plaque psoriasis.

**Methods:**

A multicenter, two-arm parallel group, single-blind, randomized, controlled trial will be conducted at six hospitals in China between August 1, 2019 and December 31, 2021. A total of 122 adult patients (aged 18–65 years) who meet the inclusion criteria are being recruited. Participants will receive either basic treatment combined with moving cupping therapy or basic treatment combined with moving cupping placebo. The treatment cycle will be 4 weeks, and the efficacy of treatment will be assessed weekly by the Psoriasis Area and Severity Index during the treatment period and follow-up visits at weeks 6 and 8. The body surface area, physician’s global assessment, Dermatology Life Quality Index, patient-reported quality of life, visual analog scale, Traditional Chinese Medication syndrome scoring scale, combined medication, and adverse events will also be recorded and compared to the relative baseline values.

**Discussion:**

The findings of this trial may lead to better decisions regarding the treatment of plaque psoriasis. If the trial outcomes are considered favorable, this ancient Chinese medical therapy may be worthy of widespread use because of its convenience and low cost.

**Trial registration:**

This study was registered on May 15,2019 at ClinicalTrials.gov with the identifier number NCT03952676.

## Background

Psoriasis is a chronic inflammatory skin disease that affects approximately 2–3% of the global population [1, 2]. It is characterized by epidermal hyperproliferation, abnormal keratinocyte differentiation, angiogenesis with blood vessel dilatation, and excess T-helper cell type 1 (Th1) and Th17 inflammation [3]. The plaque type is the most common form of psoriasis, accounting for 85–90% of psoriasis cases [1]; it leads to detrimental physical effects and reduced psychological well-being [4, 5]. It is also closely related to metabolic syndrome, cardiovascular disease, and chronic obstructive pulmonary disease [6]. For most patients, psoriasis results in the restriction of various aspects of everyday life, enormous personal costs, and mental stress [7].

The current treatment for psoriasis primarily comprises local and systemic treatments. Local treatment mainly comprises hormonal drugs and calcineurin inhibitors, and systemic treatment comprises etretin, immunosuppressants, and biological agents [8]. However, the various side effects and high economic costs limit the clinical application of these treatments [7]. Therefore, developing or identifying safe and effective treatments for psoriasis has major social and economic benefits.

Recently, complementary and alternative medicine (CAM) therapies have become an increasingly important area of dermatology, and cupping is becoming an important CAM therapy. Cupping is an ancient method that has been used worldwide. From ancient Egypt to the Han Dynasty in China [9, 10], and from Hippocrates in Greece to the early Islamic period [11, 12], there have been numerous descriptions of cupping treatments for various diseases. Cupping is currently used to treat a wide range of medical conditions [13, 14], and randomized controlled trials (RCTs) have confirmed the efficacy of cupping for certain pain-related diseases such as osteoarthritis of the knee and chronic low back pain [15, 16]. There are two types of cupping methods; dry and wet. Moving cupping therapy is a unique dry method that has been used as a traditional treatment for thousands of years. This method involves the application of lubricant to the body part or to the mouth of the cup and using the flashing method or the cotton sticking method to adsorb the cup to the treatment area. The cotton sticking method involves pasting cotton soaked in alcohol on the inner wall of the cup; it is then ignited and adsorbed on the treatment area, but the skin may be scalded due to the excessive dripping of alcohol. The flashing method quickly withdraws the cotton soaked in alcohol. This is a safe and common method of cupping. The physician pushes the cup by hand to move it up and down and left and right, thus causing flushing, congestion, and even ecchymosis of the skin in the treatment area [17]. Therefore, moving cupping therapy integrates the functions of warm moxibustion (which involves lighting the moxa stick and hanging it at a certain distance from the skin), cupping, scraping, massage, and drug therapy, and it has a wide range of clinical applications. This therapy has the ability to regulate immune function [18, 19], thereby improving skin tolerance [20, 21]. Psoriasis damages the epidermis, which destroys the human skin barrier composed of keratinocytes, intercellular substance, natural moisturizing factors, and sebum membranes. The Koebner phenomenon illustrates the important relationship between an impaired skin barrier and psoriasis [22, 23]. Moving cupping treatment can cause the mechanical stimulation of pulling and is beneficial for the secretion of sebaceous glands and sweat glands, and the use of lubricants can significantly improve the skin barrier function. Psoriasis is also known to be closely related to lipid metabolism [24]. Cupping therapy can upregulate anti-inflammatory lipids, downregulate the function of pro-inflammatory lipids, improve the balance of lipid metabolites, and alleviate the inflammatory response [25].

Moving cupping therapy has been widely used for the treatment of plaque psoriasis, which is safe and associated with few adverse reactions, mainly local blisters. Additionally, it has been recognized by a large number of peers and patients. One study compared the efficacy of moving cupping and narrowband ultraviolet B based on oral Chinese herbs and found that moving cupping has clear advantages, but lacked high-quality medical evidence [26]. Therefore, we designed a multicenter, prospective, single-blind, placebo-controlled, RCT to evaluate the safety and efficacy of moving cupping therapy for plaque psoriasis.

### Objectives and hypotheses

Moving cupping is widely used as a CAM therapy. The aim of this RCT is to evaluate the efficacy of moving cupping as a plaque psoriasis treatment. The hypothesis of this study is that the Psoriasis Area and Severity Index (PASI), used as the primary endpoint to demonstrate the clinical efficacy of plaque psoriasis treatment, will be decreased significantly with moving cupping treatment compared to placebo treatment [27]. Secondary endpoints, including body surface area (BSA), Physician’s Global Assessment (PGA), Traditional Chinese Medicine syndrome scoring scale (TCMSSS), and patient-reported outcomes (Dermatology Life Quality Index [DLQI] and visual analogy scale [VAS]), for the evaluation of post-treatment clinical efficacy, psychology, quality of life, and degree of pruritus will also be considered. This study complies with the relevant Standard Protocol Items: Recommendations for Interventional Trials (SPIRIT) Checklist (Supplementary file 1) [28].

## Methods

### Study design

The proposed study is designed as a prospective, multicenter, two-arm parallel group, single-blind RCT. Patients with plaque psoriasis will be randomly assigned to the treatment group (moving cupping) or the placebo group (sham moving cupping). Eligible participants will receive 4 weeks of treatment and a total of 8 weeks of follow-up (Table 1).

**Table 1:** Study schedules of enrollment, intervention, and assessment

	Study period							
	Enrollment	Allocation	Treatment period				Follow-up period	
Time point	-week_1_	0	week_1_	week_2_	week_3_	week_4_	week_6_	week_8_
Enrollment								
Eligibility screen	**×**							
Informed consent	**×**							
List other procedures	**×**							
Allocation		**×**						
Interventions:								
Basic treatment plus moving cupping therapy								
Basic treatment plus moving cupping placebo								
Assessments:								
PASI	**×**	**×**	**×**	**×**	**×**	**×**	**×**	**×**
BSA	**×**	**×**	**×**	**×**	**×**	**×**	**×**	**×**
PGA	**×**	**×**	**×**	**×**	**×**	**×**	**×**	**×**
DLQI		**×**	**×**	**×**	**×**	**×**		
VAS		**×**	**×**	**×**	**×**	**×**	**×**	**×**
TCMSSS		**×**	**×**	**×**	**×**	**×**		
Vital signs	**×**	**×**	**×**	**×**	**×**	**×**		
Routine blood test	**×**					**×**		
Blood biochemical test	**×**					**×**		
Routine urine test	**×**					**×**		
Food allergen test	**×**							
Combined medications	**×**	**×**	**×**	**×**	**×**	**×**		
Pregnancy test	**×**							
Physical examination	**×**					**×**		
Adverse event		**×**	**×**	**×**	**×**	**×**		
Serious adverse events		**×**	**×**	**×**	**×**	**×**		

BSA, body surface area; DLQI, Dermatology Life Quality Index; PASI, Psoriasis Area and Severity Index; PGA, Physician’s Global Assessment; PR-QoL, patient-reported quality of life; TCMSSS, Traditional Chinese Medicine syndrome scoring scale; VAS, visual analog scale

### Patient and public involvement

Patients were involved in the design of the RCT.

### Recruitment

**Fig. 1:**
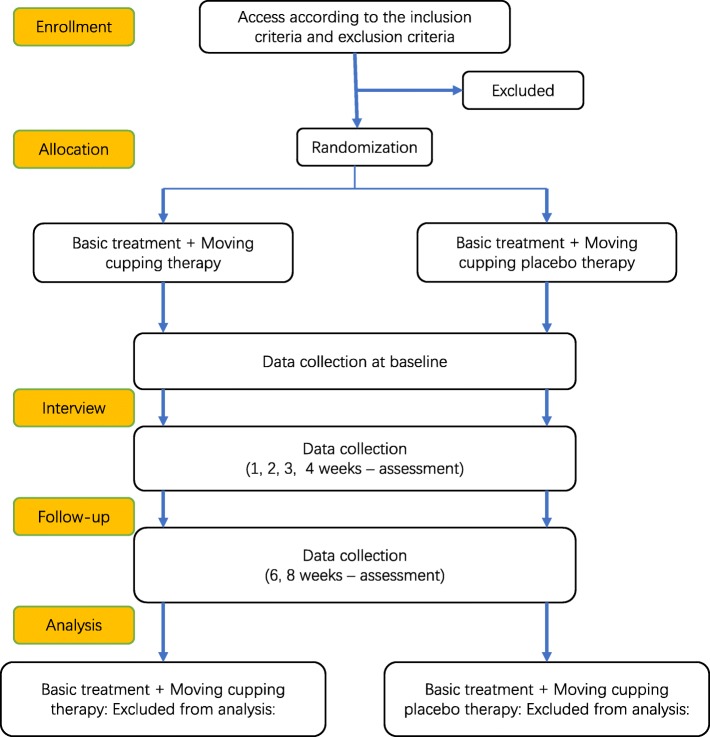
Consolidated Standards of Reporting Trials diagram of participant enrollment and analysis

We plan to recruit 122 patients with plaque psoriasis for this study. They will be evenly distributed among six hospitals using fixed enrollment. Recruitment will occur at the Yueyang Hospital of Integrated Traditional Chinese and Western Medicine (Shanghai, China), Shaanxi Traditional Chinese Medicine Hospital (Shaanxi, China), First Affiliated Hospital of Heilongjiang University of Traditional Chinese Medicine (Heilongjiang, China), the Affiliated Hospital of Jiangxi University of Traditional Chinese Medicine (Jiangxi, China), Hospital of Traditional Chinese Medicine, Xinjiang Medicine University (Xinjiang, China), and Shijiazhuang Hospital of Traditional Chinese Medicine (Shijiazhuang, China). Eligible recipients will be recruited via social media, word of mouth, and dermatology outpatient clinics at local hospitals. Each hospital will designate three dermatologists for patient recruitment and one physician (dermatologists trained and qualified to perform moving cupping) for moving cupping treatment. Patients will be informed and asked to provide written informed consent prior to study commencement. Figure 1 presents a flowchart of participant enrollment and analysis throughout the course of the trial. Researchers will obtain recipient consent prior to trial participation. All potential and enrolled recipients’ personal information will be recorded, kept in a secure folder, and made accessible only to the researchers in order to protect their privacy.

### Participant screening

All patients who are diagnosed with plaque psoriasis will undergo laboratory blood testing before inclusion, including a complete blood cell count, liver function test, renal function test, and pregnancy test. Additionally, routine urine tests, vital sign monitoring, and physical examinations will be performed (Table 1).

### Criteria

The inclusion criteria are as follows: meet the 2018 China College of Dermatology diagnostic criteria for plaque psoriasis; skin lesions involve ≤ 10% BSA (the lesions are mainly located on the torso/limbs, palm/sole, or face/scalp, with the vulva area being unaffected); aged 18–65 years; provide consent to participate in the study and sign the informed consent form.

The exclusion criteria are as follows: any clinically active skin diseases other than moderate to severe psoriasis vulgaris that might confound or influence the study aim; patients who received any systemic treatments within 4 weeks before the baseline visit; patients who received topical treatment within 2 weeks before the baseline visit (e.g., corticosteroids, ultraviolet light therapy including sunbathing); patients with an active infectious disease that is difficult to control; patients with a history of severe systemic disease, an alanine aminotransferase or aspartate transaminase level greater than 1.5-fold that of the average; any of the main routine blood indices (white blood cell count, red blood cell count, hemoglobin level, platelet count) lower than the normal limit, or other laboratory abnormalities; immunocompromised patients who may experience skin allergies or infection with moving cupping therapy; pregnant or lactating women; patients with a history of alcohol or drug abuse; and patients with a history of serious mental illness.

The exit/termination criteria were as follows: patient lost to follow-up; violation of the research plan (including lack of compliance [medication, contraindication, interview plan]); adverse events (including worsening of psoriasis above baseline [for example, PASI > 125%]) [27]; and pregnancy.

### Randomization, allocation, and blinding

Once eligibility is confirmed and consent is obtained, participants will be block-randomized using a non-stratified, permuted block with varying lengths (*n* = 6 blocks; patient distribution = 1:1) for each arm/group. Independent statisticians will provide computer-generated random sequences. Distribution will be conducted by a central web-based interactive randomization service system. When a participant is eligible, the researcher who conducts the random grouping will need to log onto the website to view the grouping situation of the participant. Due to the nature of moving cupping, it is not possible to blind the operating physician involved in treatment application. To ensure blinding, we will use a placebo cup to simulate the treatment of moving cupping and shield the patient’s eyes with a black opaque eye mask. The treatment device will be of the same size and material as well, and the treatment method will maintain consistent frequency and intensity, but with no adsorption force. Each hospital will designate one operating physician who is a dermatologist trained to perform moving cupping therapy and has obtained the relevant operational qualifications. The operating physician will not participate in the statistical analysis, and the treatment plan and grouping will be known only by the statistical analyst.

### Intervention

#### Moving cupping intervention

**Fig. 2:**
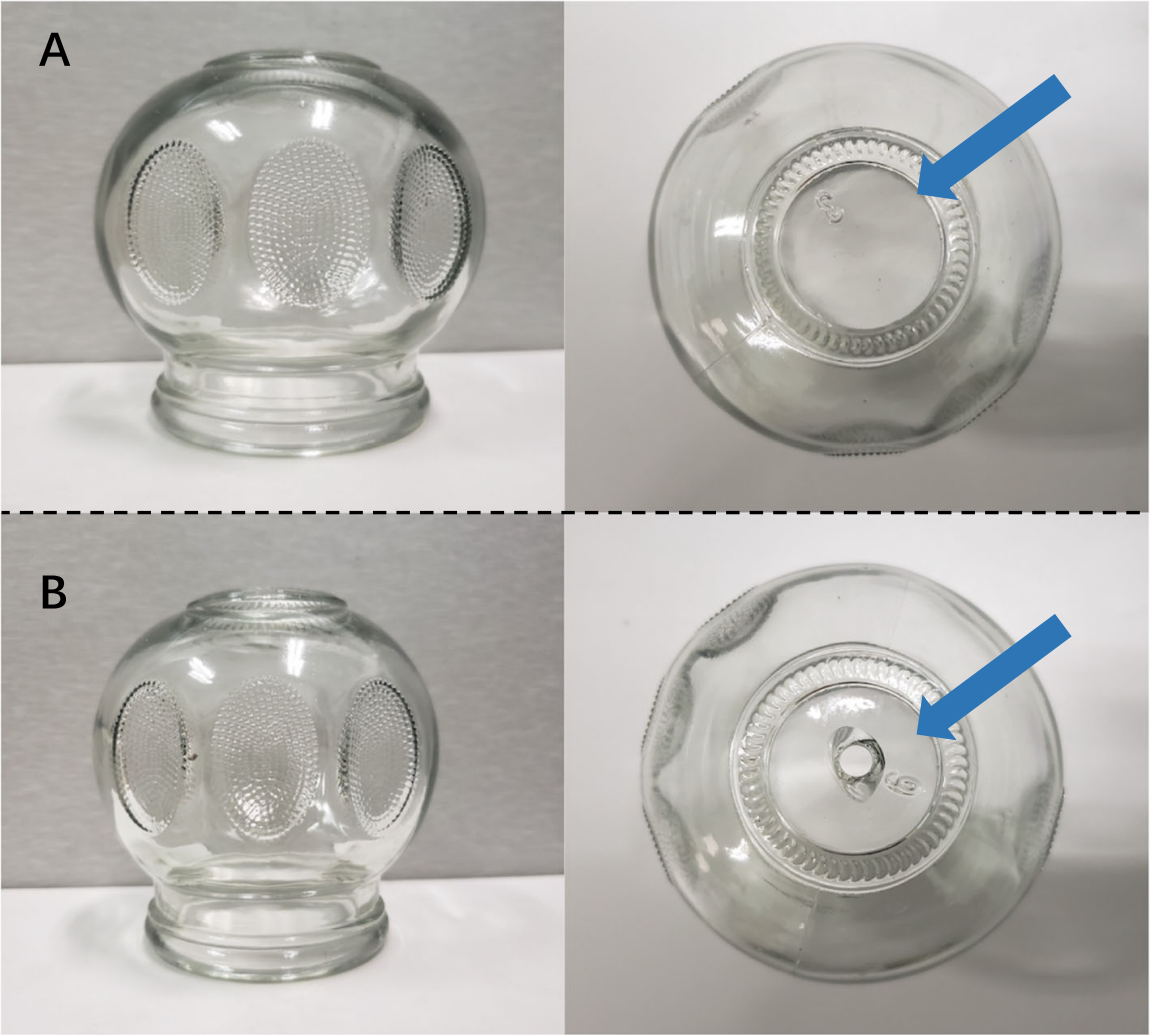
Different cups used for the intervention group and the control group when implementing moving cupping therapy. **a** The cups used for the intervention group can produce a negative pressure adsorption force when the air in the cup is burned. **b** The cup used for the placebo group has a special perforation design, and no negative pressure adsorption was formed after combustion

The cup has the texture of transparent glass. For convenience, different cup sizes will be selected according to the site of the patient’s skin lesions (Fig. 2).

Moving cupping involves standardized manipulations. First, Vaseline is applied to the skin lesion area. Using the flashing method, a 95% ethanol-soaked cotton ball is held with tweezers and the cup is held upside-down. After the cotton ball is ignited, it is immediately moved down inside the cup and removed; the cup is then quickly placed on the skin lesion area. After using the cup to absorb the skin lesion area, the cup body is held in one hand and pushed and pulled according to the skin lesion area while applying light force, such that the skin of the treatment area turns purple. Even force is applied when pushing the cup to prevent the cup from falling off due to air leakage. This is repeated on the skin lesion area 30 times [29]. The cup is changed five times per push and pull, with an interval of no more than 10 seconds. This will be performed once every other day for 4 weeks.

#### Moving cupping placebo

A special perforated cup made of the same material as that used for the intervention group will be selected and used during the same manipulation method as described for the intervention. Due to the perforated design, the fire cannot burn the air in the cup; therefore, it cannot form a negative pressure adsorption force. This simulates the form of moving cupping therapy without the therapeutic effects (Fig. 2).

#### Basic treatment

1. Increase moisturizing of the skin: As a basic treatment, the use of a moisturizer at all times is mandatory. The moisturizer should be applied to the skin areas that are free from erosion and exudation, as well as the dry non-lesion areas. A soft, fragrance-free moisturizer should be used. Yuze moisturizer will be recommended to patients with sensitive dry skin.
2. Standard bath: Generally, patients should rinse quickly with warm water (35–39 °C) once per day for approximately 5 minutes. Moisturizer should be applied within 2 minutes of bathing to avoid dehydration of the epidermis. The use of alkaline detergents to clean the skin should be avoided.
3. Avoidance of induced and aggravated factors: For some patients, certain foods can aggravate or induce disease. These foods should be avoided.
4. Some patients may have food allergies. When food allergies are identified, such foods should be avoided to prevent inducing or aggravating the condition.
5. Maintain a reasonably healthy lifestyle: Patients should avoid staying up late and becoming overly stressed. Spicy, irritating foods should be avoided, and appropriate exercise should be performed. Patients should attempt to maintain normal bowel movements.

### Measurement

#### Baseline measurements

Vital signs and disease severity will be monitored by physical examinations, biochemical examinations, and various evaluation indices before treatment. Foods that cause allergic reactions will be identified by food allergen testing. Data from these measures will be used as baseline moving cupping therapy data.

### Examination during the interview

#### Interview plan

All patients will be interviewed at weeks 1, 2, 3, and 4 during treatment. They will be reminded of the interview by phone 1 day before it is scheduled to occur. During weeks 1, 2, 3, and 4 of treatment, the psoriatic lesions will be measured by PASI, PGA, BSA, and TCMSSS, and the DLQI and VAS will be used to assess the quality of life and psychological status, and degree of pruritis, respectively. Their moisturizing, diet, and bathing habits will be monitored via patient diaries. In addition, during weeks 6 and 8 of the follow-up, the PASI, PGA, BSA, and VAS will be recorded again to evaluate the efficacy of moving cupping. Possible adverse events and combined medications should be accurately recorded at all of these time points.

### Examination during follow-up

#### Follow-up plan

All patients will be followed-up by phone at weeks 6 and 8 after treatment. During each follow-up session, the treatment effects will be assessed using the PASI, PGA, BSA, and VAS.

### Primary parameters

PASI: Incorporates the extent of psoriasis at four anatomical sites by evaluating signs of erythema, scale, and elevation. PASI scores range from 0 to 72. The primary outcome of the RCT is the proportion of patients with a reduction in PASI scores of ≥ 75% compared to baseline at the 4‑week follow-up visit [27].

### Secondary parameters

The secondary parameters of this study include the following:

1. PGA: The PGA is scored using a 5-point scale reflecting the overall degree of erythema, infiltration, and desquamation across all psoriatic lesions [27].
2. BSA: The BSA involved in psoriasis is estimated by fingerprinting, whereby the entire palm of the patient represents approximately 1% of the total BSA. The number of handprints on the psoriatic skin on a body part is used to determine the extent to which the body part is affected by psoriasis (%) [27].
3. DLQI: The DLQI is a participant-reported questionnaire used to measure the health-related quality of life of adults with skin diseases. Scores range from 0 to 30, with a higher score indicating a greater impact on the participant’s quality of life [30].
4. VAS: The VAS is used to measure lesion pruritus from 0 to 100 mm at each visit (with 0 indicating no pruritus and 100 indicating maximum pruritus) [31].
5. TCMSSS: According to the different TCM syndromes, comprehensive evaluations will be performed to determine the conditions of the tongue, pulse, and skin. The score ranges from 0 to 6 [32].

### Safety monitoring

All participants will be advised to remain under supervision in the clinical research unit for 15 minutes after treatment. In addition, data of the participants will be monitored by the research team to detect any adverse events throughout the study, such as skin damage or potential allergies. All potential adverse events will be recorded. If any participant experiences an adverse effect due to trial participation, they will receive free treatment and compensation accordingly. If any concerns of the participants are identified during screening or clinical assessment, further clinical evaluations and/or investigations will be performed immediately. If concerns are identified during the study, the participants will be withdrawn.

### Sample size

The sample size of the current trial was calculated based on the following formula [33]:

$$ {\mathrm{n}}_1={\mathrm{n}}_2=\frac{p_1\times \left(1-{p}_1\right)+{p}_2\times \left(1-{p}_2\right)}{{\left({p}_2-{p}_1\right)}^2}\times {\left({\mu}_{\alpha /2}+{\mu}_{\beta}\right)}^2 $$

According to recently published clinical trial results [27], the PASI-50 reached 62.1% (P_1_) in the study group and 35.3% in the control group (P_2_). Therefore, we assumed that the inspection level (α) was 0.05 and the power was 0.8 (β = 1-power = 0.2). For the two-sided tests, 51 participants will be required for each group. Given a 20% loss to follow-up, we expect to require 61 participants for each group. Therefore, this trial will require at least 122 participants.

### Timeline

Recruitment began in August 2019, and the intervention period will end in December 2020. Table 1 provides the schedule of enrollment, intervention, and assessment.

### Data collection and management

Each center will designate a full-time researcher, and data collection will be performed in a dermatology clinic using a unified data collection system (provided by Nanjing Ningqi Medical Technology Co., Ltd. Data Management Center, Nanjing, China). With the exception of the DLQI, R-QoL, and VAS, the evaluation indicators will be completed by data collectors. All data collectors will be trained to implement the PASI and other scales.

### Data monitoring and auditing

This study will establish the Data and Safety Monitoring Board (DSMB), which is composed of three members, including an acupuncturist, a dermatologist, and a statistician. All members must declare any conflict of interest during the trial. The DSMB will monitor the progress of the trial and review the safety and quality of the data. They will meet quarterly to review any adverse events and safety issues. All adverse events and severe adverse events will be reported to the main researchers, IRB, and DSMB within 24 hours. The primary investigator will receive the interim results and make the final decision regarding study termination.

### Statistical analysis

In this study, quantitative variables (PASI, DLQI, R-QoL, VAS) with normal distributions will be described as means and standard deviation; non-normal distribution will be summarized as median and interquartile range. Quantitative variables will be compared using a Student *t* test for normally distributed variables and the Wilcoxon rank-sum test for non-normally-distributed variables. Qualitative variables (sex, age) will be summarized as frequency and proportions, and the chi-square or Fisher exact test will be used to test the differences between qualitative variables. Quantitative variables with repeated measures will be applied with general linear models. *P* < 0.05 will be considered statistically significant. All statistical analyses will be performed with SAS version 9.4 statistical package (SAS Institute Inc., Cary, NC, USA). The measurer will be blinded to the results.

### Missing data

The possibility of loss to follow-up has been considered and will be calculated as a part of the sample size estimation. The dropout rates and reason will be recorded. In addition, we will account for other types of randomly missing data by treating dropouts as non-success or non-survival using the intention-to-treat principle.

### Ethics and dissemination

The study has been approved by the ethics committee of Yueyang Hospital of Integrated Traditional Chinese and Western Medicine (ref. approval no. 2019–003). Patients will be informed at the beginning of the study that they have the right to withdraw from the study at any time without providing a reason. Even in the event of a withdrawal, the required treatment will be provided to the patient. The results of the study will be published in an international peer-reviewed journal.

## Discussion

Psoriasis often requires multiple methods of combination therapy in the clinic; however, this does not always result in satisfactory outcomes. Therefore, there is increasing concern regarding the current combinations of CAM in modern medical practice. Cupping is a form of CAM that has existed for thousands of years in various civilizations. It plays a unique role in various diseases, including dermatology. Studies have found that cupping can lower the level of superoxide dismutase in the blood, which has a role in reducing oxidative stress [34, 35]. Cupping can also significantly reduce the hemoglobin level in the cupping area and increase the level of oxyhemoglobin. In addition, it can increase HSP-70 and β-endorphins to relieve pain [36]. Although the mechanism of moving cupping treatment for psoriasis is not clear, there are indications that moving cupping can alleviate plaque psoriatic skin inflammation and excessive thickening of skin lesions. Conventional treatment combined with moving cupping has a better curative effect. However, it is true that the therapeutic effect still requires further rigorous scientific verification.

Currently, only small-scale clinical research has been performed for moving cupping treatment of psoriasis. Relevant RCT studies did not adopt blinding methods, lacked placebo control, did not follow the CONSORT statement, and were published in languages other than English [26, 29]. Therefore, large RCTs are needed to assess the role and possible adverse outcomes of moving cupping for the treatment of plaque psoriasis.

This study will be the first placebo-controlled study of moving cupping for the treatment of plaque psoriasis with multiple centers, double-arm parallel groups, and a single-blind RCT. Furthermore, the study has several advantages. The first is that it will use a placebo control to exclude the placebo effect, reduce the risk of bias, and define the clinical efficacy of moving cupping for the treatment of plaque psoriasis. The second advantage is that, because of the large land area of China, the study will include a total of six hospitals in different regions, thus including various ethnic and socioeconomic populations. As such, its experimental design will increase the generalizability of the trial outcomes. However, this study also has some limitations. Due to the particularity of moving cupping therapy, it is difficult for different operators to maintain the same strength when moving the cup. Furthermore, because different cup sizes will be selected for the different treatment sites, adsorption forces will be inconsistent. All of these limitations may lead to the risk of bias. The placebo cupping therapy method has been used previously in only two clinical trials [37, 38]. Although the moving cupping placebo lacks absorption during treatment, we found that most patients do not understand the specific operation of the treatment method; therefore, they cannot distinguish between the moving cupping placebo and moving cupping. Patients were blinded to whether they are accepting real or placebo cupping. In conclusion, this study aims to verify whether moving cupping therapy is effective for treating plaque psoriasis. We also hope to provide a safe and effective treatment for plaque psoriasis through this study.

## Trial status

The protocol version number is 2.0 and the version date is May 15, 2019. The study plans to start recruiting participants in August 2019 and complete the recruitment in December 2020.

## Supplementary information

**Additional file 1.** SPIRIT 2013 Checklist.

**Additional file 2.**

## Data Availability

We declare that the materials described in the manuscript, including all relevant raw data, will be freely available to any scientist wishing to use them for non-commercial purposes without breaching participant confidentiality.

## Abbreviations

BSA: Body surface area
CAM: Complementary and alternative medicine
DLQI: Dermatology Life Quality Index
DSMB: Data and Safety Monitoring Board
PASI: Psoriasis Area and Severity Index
PGA: Physician’s Global Assessment
RCT: Randomized controlled trial
TCMSSS: Traditional Chinese Medicine syndrome scoring scale
Th1: T-helper cell type 1
VAS: Visual analogue scale
