# Supplementary material for: Effects of moving cupping therapy for plaque psoriasis: study protocol for a randomized multicenter clinical trial
Source: Trials. 2020 Feb 26;21:229. doi: 10.1186/s13063-020-4155-0 (PMC7045603; doi:10.1186/s13063-020-4155-0)
Supplement: Supplementary file 2 — Additional file 2. [file 13063_2020_4155_MOESM2_ESM.docx]

**PASI: Method for calculating the Psoriasis Area and Severity Index (PASI)**

Shown below is the original description of the PASI (Fredriksson T, Pettersson U. Dermatologica 1978;157:238-44) which involves the assessment of erythema (E), infiltration (I), and desquamation (D), and body surface area involvement (A) over 4 body regions (head (h), trunk (t), upper (u) and lower (l) extremities).

| Degree of severity (per body region) | Value  given |
| --- | --- |
| No symptoms | 0 |
| Slight | 1 |
| Moderate | 2 |
| Marked | 3 |
| Very marked | 4 |

| Surface involved (per body region) | Value  given |
| --- | --- |
| <10% | 1 |
| 10-29% | 2 |
| 30-49% | 3 |
| 50-69% | 4 |
| 70-89% | 5 |
| 90-100% | 6 |

Because the head, upper extremities, trunk, and lower extremities correspond to approximately 10, 20, 30, and 40% of body surface area, respectively, the PASI score is calculated by the formula:

PASI = 0.1(Eh + Ih +Dh) Ah + 0.2 (Eu + Iu + Du) Au + 0.3 (Et + It + Dt) At + 0.4 (El +Il +Dl) Al

**PGA: Example of a Psoriasis Global Assessment (PGA)**

| Severe | Very marked plaque elevation, scaling, and/or erythema | 5 |
| --- | --- | --- |
| Moderate to severe | Marked plaque elevation, scaling, and/or erythema | 4 |
| Moderate | Moderate plaque elevation, scaling, and/or erythema | 3 |
| Mild | Slight plaque elevation, scaling, and/or erythema | 2 |
| Almost clear | Intermediate between mild and clear | 1 |
| Clear | No signs of psoriasis (post-inflammatory hyperpigmentation may be present) | 0 |

**Body Surface Area (BSA)**

• BSA is a commonly used measure of severity of skin disease

• Defined as the percentage of the total body surface area affected by psoriasis

• Handprint Method

• Most common method in clinic and clinical trials

• Use patient’s full handprint to estimate 1%

**Visual Analogue Scale (VAS)**

The VAS is used to measure lesion pruritus from 0 to 100 mm at each visit (with 0 indicates no pruritus and 100 indicates maximum pruritus).


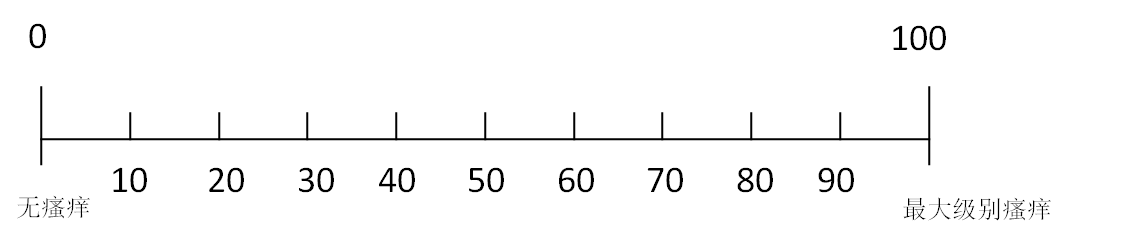

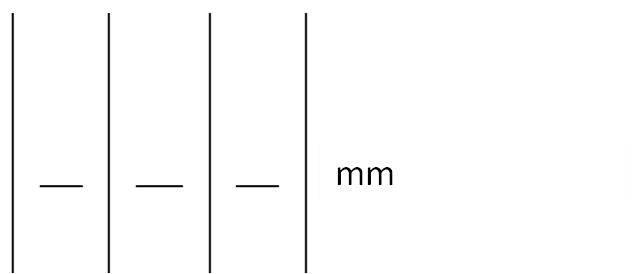


**Traditional Chinese medicine syndrome scoring scale (TCMSSS)**

| symptom | score | |
| --- | --- | --- |
| Skin lesions is crimson | NO (0) | YES (1) |
| Squam‎ous dryskin, infiltrated, and have a long course of disease | NO (0) | YES (1) |
| Dark complexion or bruising lip | NO (0) | YES (1) |
| Women's menstrual color is dark or with blood clots | NO (0) | YES (1) |
| Tongue color is peachpuff, or has petechiae | NO (0) | YES (1) |
| hesitant pulse or thin and slow pulse | NO (0) | YES (1) |
| All score |  | |

**Dermatology Life Quality Index（DLQI）**

The DLQI is a participant-reported questionnaire used to measure the health-related quality of life of adults with skin diseases. Scores range from 0 to 30, with a higher score indicating a greater impact on the participant’s quality of life. [19]

1. Over the last week, how itchy, sore, Very much 

painful or stinging has your skin A lot 

been? A little 

Not at all 

2. Over the last week, how embarrassed Very much 

or self conscious have you been because A lot 

of your skin? A little 

Not at all 

3. Over the last week, how much has your Very much 

skin interfered with you going A lot 

shopping or looking after your home or A little 

garden? Not at all  Not relevant 

4. Over the last week, how much has your Very much 

skin influenced the clothes A lot 

you wear? A little 

Not at all  Not relevant 

5. Over the last week, how much has your Very much 

skin affected any social or A lot 

leisure activities? A little 

Not at all  Not relevant 

6. Over the last week, how much has your Very much 

skin made it difficult for A lot 

you to do any sport? A little 

Not at all  Not relevant 

7. Over the last week, has your skin prevented Yes 

you from working or studying? No  Not relevant 

If "No", over the last week how much has A lot 

your skin been a problem at A little 

work or studying? Not at all 

8. Over the last week, how much has your Very much 

skin created problems with your A lot 

partner or any of your close friends A little 

or relatives? Not at all  Not relevant 

9. Over the last week, how much has your Very much 

skin caused any sexual A lot 

difficulties? A little 

Not at all  Not relevant 

10. Over the last week, how much of a Very much 

problem has the treatment for your A lot 

skin been, for example by making A little 

your home messy, or by taking up time? Not at all  Not relevant 
